# Gray Wolf Optimization-Long Short-Term Memory Based Temperature Estimation and Closed-Loop Control Method in Microfluidic Chemiluminescence Immunoassay

**DOI:** 10.3390/mi17070803

**Published:** 2026-06-30

**Authors:** Xu Xu, Zhongyi Xu, Chuan Lyu, Bo Liang, Congcong Zhou, Xuesong Ye, Jing Wang

**Affiliations:** 1 College of Biomedical Engineering and Instrument Science, Zhejiang University, Hangzhou 310027, China; 2 Sir Run Run Shaw Hospital, School of Medicine, Zhejiang University, Hangzhou 310016, China

**Keywords:** temperature estimation, GWO-LSTM model, microfluidic chip, chemiluminescence immunoassay

## Abstract

Driven by the rising demand for point-of-care testing (POCT) in aging societies, accurate temperature regulation of reaction solutions has become a core technical bottleneck for miniaturized chemiluminescence immunoassay systems, since conventional indirect control strategies inevitably produce systematic deviations. To tackle this challenge, we present an integrated solution that couples multiphysics simulation, data-driven temperature estimation modeling, and embedded hardware design. We constructed a COMSOL heat transfer model to analyze the thermal performance of the microfluidic chip. Meanwhile, a grey wolf optimization (GWO) enhanced long short-term memory (LSTM) network was developed to infer the unmeasured actual reaction solution temperature based on accessible parameters, including heating voltage, ambient temperature and substrate temperature. The obtained temperature estimation was then fed back to a fuzzy PID controller for closed-loop regulation. Experimental results demonstrated that the GWO-LSTM model limited the estimation error within 0.3 °C, and the steady-state temperature control accuracy reached ±0.2 °C or higher under fluctuating ambient conditions and diverse initial states. For cardiac troponin I (cTnI) detection, the proposed system shortened the incubation duration and reduced the coefficient of variation from 10.77% to 2.69%. This work addresses the key bottleneck restricting precise temperature control in microfluidic chemiluminescence analyzers, which provides robust technical support for the development of next-generation high-performance POCT instruments.

## 1. Introduction

With the accelerating aging of the population and the growing demand for chronic disease management, the development of high-precision, miniaturized point-of-care testing (POCT) devices has become increasingly critical [1,2,3,4]. Integrating chemiluminescence immunoassay technology with microfluidic chips provides an ideal approach to advancing high-performance POCT systems. However, achieving direct and precise temperature control of the chemiluminescent reaction solution in miniaturized platforms remains a key technical bottleneck. Conventional indirect temperature measurement methods inherently introduce control deviations in miniaturized systems, which, in turn, limit the analytical performance of microfluidic chemiluminescence immunoassays.

The incubation temperature of the reaction solution serves as a critical determinant in chemiluminescence immunoassays, and its precise regulation directly dictates the efficiency and specificity of the immunoreaction, as well as the accuracy and stability of the analytical outcomes [5,6,7,8]. An optimal incubation temperature ensures efficient and specific antigen–antibody binding, accelerates reaction kinetics, and facilitates the formation of antigen–antibody complexes, thereby enhancing chemiluminescent (CL) intensity and lowering the detection limit [9]. In contrast, excessively low temperatures significantly decelerate the immunoreaction, leading to incomplete antigen–antibody binding and attenuated signal output, which may result in false-negative results or diminished analytical sensitivity. Excessively high temperatures, on the other hand, can disrupt the conformational integrity and biological activity of both antigens and antibodies, induce nonspecific binding events, elevate background signals, and compromise assay specificity. Furthermore, elevated temperatures may adversely impact the reaction efficiency of the chemiluminescent substrate, potentially causing measurement bias or even complete assay failure. Additionally, even minor fluctuations in incubation temperature can directly undermine the repeatability of the assay. Therefore, maintaining precise and stable incubation conditions is indispensable to ensuring that chemiluminescence immunoassays achieve high sensitivity, specificity, and robust intra- and inter-assay repeatability in clinical diagnostic settings—thus meeting the critical requirement for accurate quantitative analysis in disease diagnosis [10].

To enhance the temperature control accuracy of microfluidic systems, researchers have explored strategies for the direct measurement of liquid temperature through the integration of temperature sensors into microfluidic chips [11]. Specifically, Lee et al. developed an integrated microfluidic platform capable of achieving precise temperature control within ±0.25 °C over the range of 25 °C to 95 °C, thus demonstrating excellent thermal regulation performance for various biochemical reaction scenarios [12,13]. In a separate study, Meng et al. employed millimeter-scale industrial temperature sensors for microscale liquid temperature sensing, which exhibited a dynamic linear measurement range of 30 °C to 100 °C with a measurement uncertainty of less than 0.5 °C [14]. Despite the promising temperature sensing performance of this direct measurement approach, it has a critical practical limitation: sensor materials must be used as consumables, which inevitably increases the overall operational costs and impedes its large-scale clinical and industrial application.

In alternative research efforts, temperature-responsive functional materials have been incorporated into reaction systems to enable liquid temperature deduction and regulation through optical intensity monitoring [15,16,17]. This strategy is designed to alleviate detection errors stemming from the thermal hysteresis between the internal solution of the reaction chamber and the heating module. In a representative study, Geitenbeek et al. exploited the thermally coupled energy levels of Er^3+^ doped in NaYF_4_ nanoparticles to realize precise thermal regulation, achieving a temperature control resolution of 0.5 °C [17]. Nevertheless, such approaches entail rigorous reagent criteria and require costly auxiliary temperature sensing hardware. These inherent drawbacks substantially restrict their practical translation and widespread adoption in clinical scenarios.

Recent advances have demonstrated that real-time, low-cost and noninvasive estimation of hard-to-measure physical variables can be realized by constructing mathematical correlations between easily detectable parameters and target quantities. Meng et al. presented an indirect temperature sensing method based on liquid metal heat transfer theory. By establishing a correlation between commercial industrial temperature sensors and the thermal field of on-chip fluidics, their approach enables precise microfluidic temperature prediction via a well-constructed heat transfer model [14]. In the context of PCR thermal control systems, Wang et al. adopted a system identification framework to develop dedicated mathematical models for the heating and cooling phases separately, and further constructed a virtual sensor based on a model-switching strategy. When integrated with a fuzzy PID controller, the established virtual sensor effectively improved the closed-loop temperature control accuracy of the reaction solution to ±0.21 °C [18].

However, temperature estimation relying on system modeling still suffers from notable inherent limitations. Generally, such models are established based on predefined physical principles, empirical correlations, or experimental datasets, inevitably introducing simplifications and idealized assumptions when characterizing internal heat transfer mechanisms, as well as external environmental interferences. Accordingly, they fail to faithfully replicate the intricate thermal transfer behaviors under practical operational scenarios. Furthermore, model parameters are highly vulnerable to non-ideal interference factors, including ambient temperature and humidity fluctuations, as well as variable thermal contact resistance, which readily induce parameter drift over time. In addition, it remains challenging to fully incorporate all stochastic disturbances and inherent nonlinear characteristics throughout the modeling procedure. As such, conventional models exhibit insufficient adaptability and robustness under dynamically varying operating conditions, inevitably giving rise to temperature estimation deviations. Any alteration in system configuration or working environment necessitates tedious model recalibration, further restricting the method’s generalization capability and real-time implementation performance. Collectively, these drawbacks severely hinder traditional system-modeling-based strategies from meeting the stringent demands of high-precision and highly dynamic temperature estimation. The pure physical-model-based virtual sensing strategy [14] and the system-identification-based fuzzy PID control [18] have achieved promising temperature regulation performance. Nevertheless, these studies still suffer from inherent defects: Ref. [14] depends heavily on idealized heat transfer equations, which are accurate but less suitable for routine disposable microfluidic assay chips and are susceptible to parameter drift and environmental disturbances under dynamic working conditions; Ref. [18] adopts conventional system identification and model-switching control, which lacks the capability to handle complex nonlinear thermal variations and stochastic fluctuations in microfluidic systems.

This study proposes an integrated framework that combines multiphysics simulation, temperature estimation, and intelligent control algorithms. First, finite element simulation is conducted via COMSOL 6.1 to construct a multiphysics heat transfer model, which enables precise characterization of the thermodynamic properties of the reaction solution system inside the microfluidic chip. On this basis, a novel finite element data-driven temperature estimation model is established by leveraging a grey wolf optimization (GWO)-enhanced long short-term memory (LSTM) network. By extracting the time-series characteristics of easily measurable parameters such as heating voltage, ambient temperature, and thermal substrate temperature, the developed model achieves high-precision real-time estimation of the actual reaction solution temperature, which cannot be directly measured physically. Furthermore, the estimated temperature is adopted as the feedback signal and combined with a fuzzy PID controller to formulate an innovative intelligent temperature control system. Compared with the sensor-dependent, pure-physics-based [14], and traditional identification-based strategies [18], the proposed hybrid simulation-machine learning-control framework effectively suppresses model drift, improves environmental adaptability and nonlinear fitting ability, and achieves higher accuracy and stronger robustness for dynamic microfluidic temperature regulation.

## 2. Materials and Methods

### 2.1. Microfluidic Chip

The microfluidic chip body, as well as its upper and lower cover films, is fabricated from fluorinated ethylene propylene (FEP), a material characterized by excellent biocompatibility and chemical inertness, resulting in an integrated, compact structural layout.

The chip is configured with five sequentially arranged, independent functional chambers. The first four chambers each have dimensions of 6.5 mm × 5 mm × 6 mm, which are preloaded with reagents and allocated for distinct reaction procedures. The fifth chamber serves as a relatively miniaturized substrate chamber with dimensions of 4 mm × 4 mm × 6 mm, dedicated to the storage of chemiluminescent substrates. A microchannel gap of approximately 0.1 mm is precisely reserved between the bottom of each partition wall (that separates adjacent chambers) and the basal film of the chip.

### 2.2. MDMF-CLIA Analyzer

**Figure 1:**
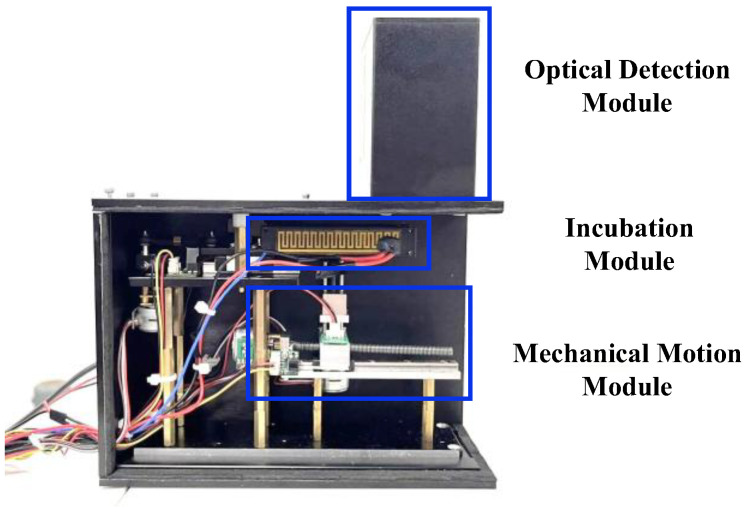
Structure diagram of the magnetically driven MDMF-CLIA analyzer.

As shown in Figure 1, the magnetically driven microfluidic chemiluminescence immunoassay (MDMF-CLIA) analyzer features overall external dimensions of 16 cm × 6 cm × 18 cm. The system is mainly composed of three core functional units: an incubation module, a mechanical motion module, and an optical detection module. The incubation module is arranged on both sides of the chip placement region, which provides precise thermal regulation for reaction chambers after chip loading to facilitate the progression of antigen–antibody binding reactions.

The magnetic separation and washing module consists of a magnetic control unit and a mixing unit [19]. The magnetic control unit adopts neodymium–iron–boron (NdFeB) permanent magnets (N52 grade, approximately 1.42 T, 10 mm × 10 mm × 10 mm), which are arranged in an alternating north–south pole configuration. Driven by a stepper motor, the module achieves precise horizontal (*x*-axis) and vertical (*z*-axis) displacement, enabling the transfer, controlled aggregation and dispersion of magnetic beads across adjacent chambers. The mixing unit drives the chip to generate periodic lateral vibration, thereby improving the uniform dispersion of magnetic beads within the reaction solution.

Integrated directly above the substrate chamber, the optical detection module employs a photomultiplier tube (PMT, Model CH345, Beijing Hamamatsu Photon Techniques Co., Ltd., Beijing, China) to capture chemiluminescence signals emitted from the immunoreaction between magnetic immunocomplexes and the substrate [20]. The collected optical signals are subsequently converted into electrical signals for subsequent quantitative analysis. The entire device is encapsulated in a black aluminum enclosure to suppress ambient light interference. Meanwhile, an STM32F407ZET6 microcontroller is adopted as the central control unit, realizing full-process automated operation of the system.

### 2.3. Incubation Module Modeling and Co-Simulation Framework

This study focuses on the incubation module integrated into the magnetically driven microfluidic chemiluminescence immunoassay system.

The module mainly comprises a microfluidic chip, a thermal substrate, resistive heating components, and temperature sensors, which collectively maintain a stable thermal environment for the reaction solution throughout the immunoreaction process. The thermal substrate acts as the carrier for chip placement and guarantees uniform temperature regulation. The heating unit adopts two polyimide heating films (10 mm × 50 mm), symmetrically affixed to both sides of the thermal substrate for precise temperature modulation. Temperature monitoring is implemented via a PT1000 platinum resistance sensor (Kaipusen (Taizhou) Measurement and Control Technology Co., Ltd, Taizhou, China), which features a fast transient response to capture subtle temperature fluctuations and deliver reliable feedback signals. Given that both antigen–antibody binding kinetics and enzymatic reaction efficiency are highly temperature-dependent, the dynamic response capability and steady-state temperature precision of the incubation module directly determine the overall analytical performance of the system.

**Figure 2:**
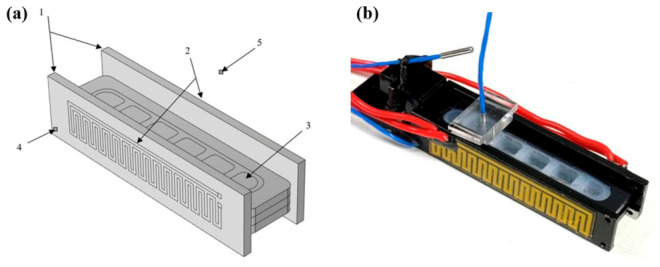
Structure diagram of the incubation module: (**a**) Model diagram: 1 is the thermal substrate, 2 is the heating film, 3 is the reaction solution, 4 is the temperature sensor in the thermal substrate, 5 is the ambient temperature sensor. (**b**) The corresponding physical device diagram.

To elucidate the heat transfer characteristics of the incubation module and lay a theoretical foundation for subsequent control strategy development, a multiphysics finite element model of the module was established, as illustrated in Figure 2. Numerical modeling was implemented on the COMSOL Multiphysics platform. By assigning diverse ambient temperature boundary conditions, the temperature field distribution and transient thermal response behavior of the system during the heating process were systematically explored.

In the modeling procedure, the geometric configuration of the heating film was first defined. The embedded nickel–chromium resistive layer inside the heating film was parameterized as a serpentine structure, with a thickness of 10 μm and a width of 8 mm. Two silver contact pads (10 mm × 10 mm × 10 μm) were configured at the external lead terminals to facilitate electrical connection. The thermal substrate was simplified as a bulk aluminum solid domain in the model to faithfully characterize its intrinsic thermal properties.

To simulate heat transfer behavior within the thin conductive layer, the model concurrently employed the Heat Transfer in Solids interface (from the Heat Transfer Module) and the Electric Currents, Layered Shell interface (from the AC/DC Module) of COMSOL Multiphysics. On the electrical side, the Electric Currents, Layered Shell interface was utilized to model the Joule heating effect induced by electrical conduction. In the simulation, an electrical potential U1 was applied to one edge of the first silver contact pad, while one edge of the second silver contact pad was grounded to establish the required applied voltage boundary condition. The multiphysics coupling mechanism was defined as electromagnetic heating to realize the synergistic simulation of electrical conduction and heat transfer.

The surface heat rate generated within the thin layer (W/m^2^) is expressed as follows:

(1) $$q_{prod}=dQ_{DC}$$

where *Q_DC_* denotes the Joule heat source derived from the current conservation equation (W/m^3^) and *t* represents the thickness of the conductive layer. Owing to its relatively high electrical resistivity, the nickel–chromium alloy layer serves as the primary heat source. The generated heat is transferred to the thermal substrate of the heating film in the form of inward heat flux. The volumetric power density is defined as:

(2) $$Q_{DC}=J\cdot E=\sigma {\left|\nabla_{t}V\right|}^{2}$$

where *σ* is the electrical conductivity (S/m), ∇ denotes the in-plane gradient operator defined for the shell domain, and *V* represents the electric potential.

Convective and radiative cooling boundary conditions were applied to the external surfaces, governed by:

(3) $$n\left(-k\cdot \nabla T\right)=h_{conv}\left(T-T_{amb}\right)+\varepsilon \sigma_{sb}\left(T^{4}-{T_{amb}}^{4}\right)$$

where *n* is the outward unit normal vector at the boundary, *h_conv_* is the convective heat transfer coefficient (W/(m^2^·K)), *ε* is the surface emissivity, *σ_sb_* is the Stefan–Boltzmann constant, and *T_amb_* is ambient temperature. The parameters of the materials for each part in the COMSOL model are listed in Table 1.

**Figure 3:**
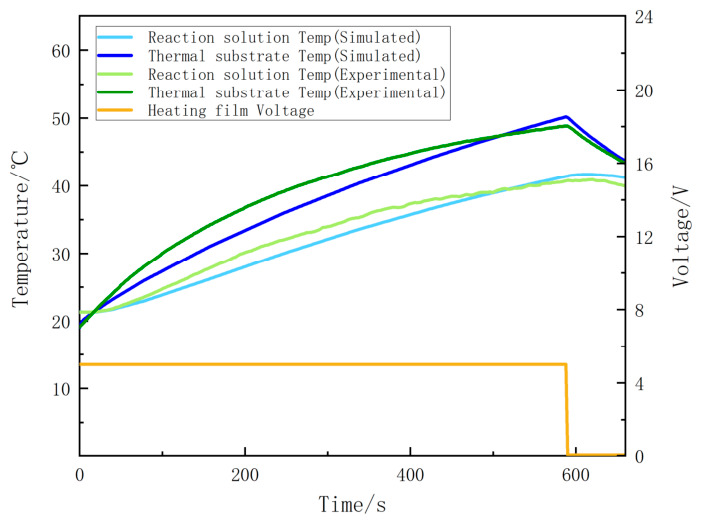
Simulation and experimental results for the heating process.

By utilizing the probe function integrated in COMSOL Multiphysics, key parameters during the heating process of the magnetically driven microfluidic chip can be directly extracted from the simulation results, including the temperature of the reaction solution inside the microfluidic chip, the temperature of the thermal substrate, and the applied voltage. To verify the reliability of the established finite element model, a comparative analysis was performed between the simulated heating performance of the microfluidic chip and the experimentally measured data. The detailed comparison results between the simulation and experiment are presented in Figure 3.

The simulated heating results exhibit a good agreement with the in situ experimental measurements, as summarized in Table 2. From the comparative analysis, the simulated temperature variation trends of both the reaction solution and the thermal substrate are highly consistent with the experimental data. Thus, the established finite element model is deemed accurate and reliable, which can be further employed for subsequent heat transfer characteristic analysis and control strategy design.

### 2.4. Reaction Solution Temperature Estimation and Optimization

Considering that, in practical systems, temperature sensors are typically embedded within or mounted on the surface of the thermal substrate and cannot directly contact the reaction solution, a GWO-LSTM temperature estimation algorithm is proposed to acquire real-time temperature information about the reaction solution during the control process. First, a high-fidelity dataset was generated using the validated finite element model under varying ambient temperature and heating power conditions. Specifically, ambient temperature, thermal substrate temperature, and heating power were defined as input variables, while the actual reaction solution temperature was designated as the output variable.

The temperature variation in microfluidic chips is a typical long time-series prediction problem. Recurrent neural networks generally exhibit stronger capability than traditional machine learning models, such as Random Forest and XGBoost, in capturing temporal dependencies. Among recurrent architectures, LSTM is particularly suitable for modeling long-range temporal correlations and alleviating the gradient vanishing problem compared with GRU. Therefore, LSTM was adopted as the baseline model in this work, and the main focus was placed on improving its prediction performance through GWO-based hyperparameter optimization rather than conducting extensive comparisons with other models.

The fundamental principle of the LSTM network lies in regulating the information flow via a sophisticated gating mechanism, which enables the effective capture of long-term dependencies inherent in time-series data. The LSTM architecture comprises an input gate, a forget gate, an output gate, and a cell state. Through these gated units, the network dynamically decides, at each time step, the amount of information to retain, discard, or output, thereby achieving robust processing of time-series data with long-term temporal correlations.

The forget gate evaluates the current input *x_k_* and the previous hidden state *h_k_*_−1_, and employs a sigmoid activation function with an output range of [0, 1] to regulate the internal state. It determines the degree to which the previous memory *c_k_*_−1_ is preserved. The forget gate output *f_k_* is computed as:

(4) $$f_{k}=\sigma \left(W_{f}\cdot \left[x_{k},h_{k-1}\right]+b_{f}\right)$$

where *σ* denotes the sigmoid function, *W_f_* represents the weight matrix of the forget gate, and *b_f_* is the corresponding bias term.

The input gate similarly processes *x_k_* and *h_k_*_−1_ through a sigmoid function to determine the update magnitude *i_k_*. Meanwhile, the same inputs are passed through a hyperbolic tangent function to generate a candidate memory ${\hat{c}}_{k}$. The updated cell state is obtained by combining the forget and input mechanisms as follows:

(5) $$i_{k}=\sigma \left(W_{i}\cdot \left[x_{k},h_{k-1}\right]+b_{i}\right)$$

(6) $${\hat{c}}_{k}=\tanh\left(W_{c}\cdot \left[x_{k},h_{k-1}\right]+b_{c}\right)$$

(7) $$c_{k}=f_{k}\cdot c_{k-1}+i_{k}\cdot {\hat{c}}_{k}$$

where *W_i_* and *b_i_* denote the input gate weights and bias, respectively; *W_c_* and *b_c_* are the parameters associated with internal state updates.

The output gate determines the degree to which the updated cell state contributes to the hidden state. The hidden state *h_k_* is calculated as:

(8) $$o_{k}=\sigma \left(W_{o}\cdot \left[x_{k},h_{k-1}\right]+b_{o}\right)$$

(9) $$h_{k}=o_{k}\cdot \tanh\left(c_{k}\right)$$

where *W_o_* and *b_o_* are the output gate weight matrix and bias term, respectively.

To address the limitation that baseline LSTM models heavily rely on manual hyperparameter tuning, the GWO algorithm was introduced. Specifically, the key hyperparameters of the LSTM model—including the number of hidden units, initial learning rate, number of LSTM layers, and dropout rate—were encoded as position vectors of grey wolf individuals within the predefined search space.

GWO simulates the social hierarchy and hunting mechanism of grey wolves. The population is organized into four hierarchical levels: α, β, δ, and ω wolves. The α wolf represents the best candidate solution and leads the search process; β and δ wolves assist in guiding the search, while ω wolves occupy subordinate positions.

During the encircling phase, wolves update their positions relative to the prey according to:

(10) $$\vec{D}=\left|\vec{C}\cdot \vec{X_{P}}\left(t\right)-\vec{X}\left(t\right)\right|$$

(11) $$\vec{X}\left(t+1\right)=\vec{X_{P}}\left(t\right)-\vec{A}\cdot \vec{D}$$

where *t* denotes the iteration index, $\vec{X_{P}}\left(t\right)$ is the prey position vector, $\vec{D}$ represents the random weight indicating the impact of the wolf’s position on the prey, and $\vec{X}\left(t\right)$ represents the grey wolf position vector. The coefficient vectors $\vec{A}$ and $\vec{C}$ are defined as:

(12) $$\vec{A}=2\vec{\alpha }\cdot \vec{r_{1}}-\vec{\alpha },\vec{C}=2\vec{r_{2}}$$

where $\vec{\alpha }$ decreases linearly from 2 to 0 over the course of iterations, and $\vec{r_{1}}$, $\vec{r_{2}}$ are random vectors within [0, 1].

In the hunting phase, grey wolves are capable of identifying and encircling prey. The hunting process is typically guided by the α wolf, while β and δ wolves may also participate in decision-making. In an abstract search space, the wolves do not possess explicit knowledge of the exact position of the optimal solution. To simulate this mechanism, it is assumed that the α (best candidate solution), β, and δ wolves have superior knowledge regarding the potential location of the prey. Therefore, during each iteration, the three best solutions obtained thus far are retained. The remaining wolves (including ω) update their positions based on the guidance of these leading individuals according to the following formulation:

(13) $$\left\{\begin{array}{l} \vec{D_{\alpha }}=\left|\vec{C_{1}}\cdot \vec{X_{\alpha }}-\vec{X}\left(t\right)\right|\\ \vec{D_{\beta }}=\left|\vec{C_{2}}\cdot \vec{X_{\beta }}-\vec{X}\left(t\right)\right|\\ \vec{D_{\delta }}=\left|\vec{C_{3}}\cdot \vec{X_{\delta }}-\vec{X}\left(t\right)\right| \end{array}\right.$$

(14) $$\left\{\begin{array}{l} \vec{X_{1}}=\vec{X_{\alpha }}\left(t\right)-\vec{A_{1}}\cdot \vec{D_{\alpha }}\\ \vec{X_{2}}=\vec{X_{\beta }}\left(t\right)-\vec{A_{2}}\cdot \vec{D_{\beta }}\\ \vec{X_{3}}=\vec{X_{\delta }}\left(t\right)-\vec{A_{3}}\cdot \vec{D_{\delta }} \end{array}\right.$$

(15) $$\vec{X}\left(t+1\right)=\frac{\vec{X_{1}}+\vec{X_{2}}+\vec{X_{3}}}{3}$$

where $\vec{X_{\alpha }}$, $\vec{X_{\beta }}$ and $\vec{X_{\delta }}$ denote the position vectors of the α, β, and δ wolves at the current iteration, respectively; $\vec{X}\left(t\right)$ represents the position vector of an individual wolf at iteration *t*; $\vec{C_{1}}$, $\vec{C_{2}}$, and $\vec{C_{3}}$ are random coefficient vectors; $\vec{D_{\alpha }}$, $\vec{D_{\beta }}$ and $\vec{D_{\delta }}$ represent the distances between the current individual and the α, β, and δ wolves; and $\vec{X}\left(t+1\right)$ is the updated position vector.

During the final stage of hunting, once the prey ceases movement, the wolves converge to attack and capture it, which corresponds to determining the optimal solution. This process is primarily achieved through the gradual reduction in the convergence factor a during iterations, resulting in a linear decrease in the fluctuation range of the coefficient vector $\vec{A}$. Consequently, the population progressively transitions from global exploration to local exploitation and converges toward the optimal solution at the end of the iteration process. The stochastic vector $\vec{C}$ plays a critical role in preventing premature convergence to local optima, thereby enhancing the global search capability, robustness, and convergence performance of the algorithm.

The overall optimization framework comprises two sequential stages. The first stage involves the GWO-based hyperparameter optimization process. Under the constraints of computational resources, the GWO algorithm is run for a predefined number of generations, and the individual with the highest fitness value during the entire search process is recorded as the optimal hyperparameter combination. The second stage corresponds to the final model training phase. Utilizing the optimal hyperparameters obtained from the first stage, the LSTM network is reconstructed and fully trained on the complete training dataset. To prevent overfitting and ensure the stability of model convergence, early stopping and model checkpoint strategies are adopted, which ultimately yields a deployable GWO-LSTM temperature estimation model.

### 2.5. Experimental Setup and Evaluation Protocol

Following the design of the control strategy and temperature estimation methodology, the proposed algorithms were deployed into the practical control system of the incubation module. The experimental platform was composed of an embedded controller, temperature sensors, a heating drive circuit, and a data acquisition module. A series of controlled comparative experiments were conducted to quantitatively evaluate key performance indicators, including heating rate, steady-state temperature error, and environmental adaptability, under different temperature control strategies.

In addition, cardiac troponin I (cTnI), a representative cardiac biomarker, was selected for practical incubation experiments. The impact of the proposed temperature control system on immunoassay performance was comprehensively assessed by analyzing the stability of the chemiluminescent signals and the coefficient of variation (CV). The magnetic beads used in the experiments were purchased from Beijing Boruimai Biotechnology Co., Ltd. (Beijing, China), while the remaining reagents were sourced from Shenzhen Junhe Biotechnology Co., Ltd. (Shenzhen, China).

## 3. Results and Discussion

### 3.1. Accuracy of Reaction Solution Temperature Estimation

To evaluate the performance of the baseline LSTM model and the GWO-LSTM model under conditions simulating practical online deployment, an autoregressive prediction scheme was adopted. In this mode, the model continuously performs predictions by feeding its previous estimated output back as part of the subsequent input.

The temperature estimation results indicate that, compared with conventional system identification methods, the LSTM model can effectively capture the dynamic variation trend of the reaction solution temperature, although non-negligible errors persist under certain operating conditions. After introducing the GWO algorithm for hyperparameter optimization, the GWO-LSTM model achieved further improvements in both prediction accuracy and stability, which significantly reduced the estimation error of the reaction solution temperature. The detailed hyperparameter optimization results of the GWO algorithm are summarized in Table 3.

**Figure 4:**
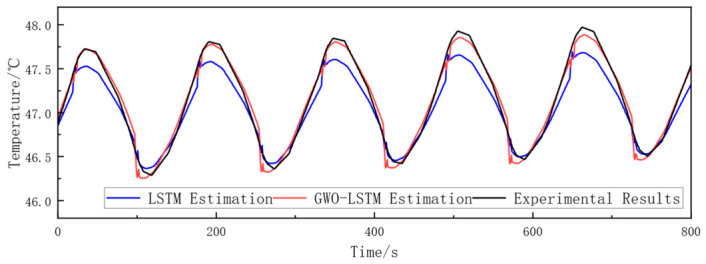
Reaction solution temperature estimation: experimental results vs. LSTM estimation and GWO-LSTM estimation.

Specifically, the GWO-LSTM model exhibited consistent performance improvements across three key evaluation metrics: mean absolute error (MAE), root mean square error (RMSE), and coefficient of determination (R^2^). As illustrated in Figure 4, the predicted temperature trajectory showed a closer alignment with the ground truth, especially at inflection points where temperature variations were more prominent, demonstrating a faster dynamic response and reduced prediction lag. Compared with the baseline LSTM model, the GWO-LSTM model reduced the MAE from 0.30 °C to 0.13 °C, the MSE from 0.12 °C to 0.03 °C, and the RMSE from 0.35 °C to 0.17 °C, while the R^2^ value increased from 93.86% to 99.64%. These results are summarized in Table 4 and demonstrate that the GWO algorithm successfully identified a more optimal combination of network capacity and regularization parameters, thereby enhancing the model’s nonlinear fitting capability without inducing overfitting.

**Figure 5:**
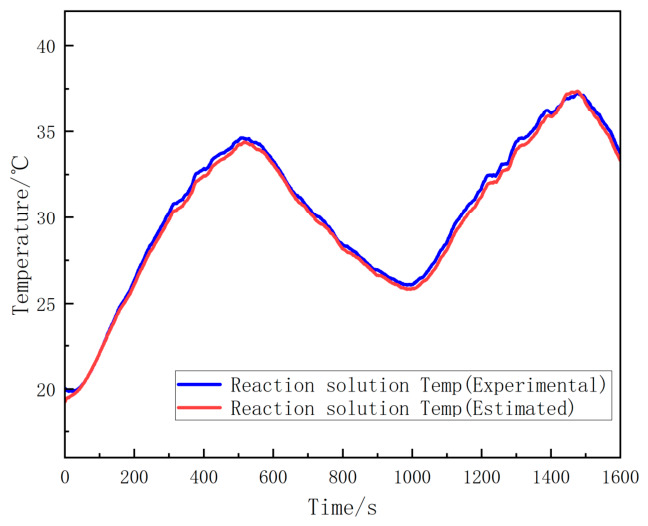
Comparison between the experimental and estimated temperatures of the reaction solution.

To further validate the real-time estimation accuracy of the GWO–LSTM model deployed on the STM32F4 platform, experimental tests were conducted under practical operating conditions. The reference ground truth was acquired using a high-precision platinum resistance patch sensor, which was positioned at the central point of the reaction solution within the microfluidic chip. Temperature data were sampled at 1 s intervals, and the real-time estimated values generated by the embedded GWO–LSTM model were compared with the measured reference values. The MAE, MAPE, RMSE, and R^2^ were calculated across all test points. The detailed experimental results are presented in the corresponding Figure 5.

During the testing process, the MAE between the model’s estimated values and the ground truth was 0.30 °C, MSE was 0.11 °C, and R^2^ reached 98.90%. The primary estimation deviations occurred during the dynamic cooling phase, which was associated with abrupt power variations. Overall, the experimental results demonstrate that the embedded virtual sensor can reliably and accurately reflect the true temperature of the reaction solution.

Specifically, during the temperature-validation experiments, a miniature calibrated platinum resistance sensor was inserted into the reaction solution phase of a dedicated validation chip to obtain the actual reaction-solution temperature. The sensing tip was positioned near the geometric center of the reaction chamber, and the lead wire was routed along the chamber wall and sealed to reduce leakage, flow disturbance, and interference with magnetic-bead motion. This inserted sensor was used only to establish the experimental ground truth for model validation and temperature-control performance evaluation; its signal was not used as feedback by the fuzzy PID controller. In practical assay operation, especially in the subsequent cTnI chemiluminescence experiments, no sensor was inserted into the reaction solution, and the controller relied entirely on the GWO-LSTM-estimated reaction solution temperature.

### 3.2. Integrated Closed-Loop Control Performance Under Different Ambient Temperatures

The GWO-LSTM temperature estimation model was integrated as the core virtual thermometer module into the control architecture, thereby forming a complete and executable temperature control system. Closed-loop performance simulations were carried out in the Python 3.12 environment to evaluate the system’s control effectiveness.

**Figure 6:**
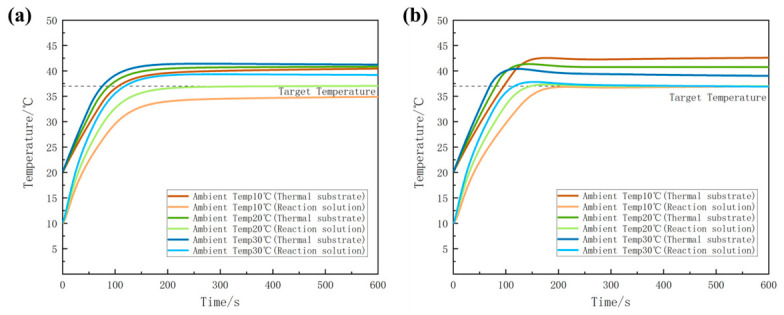
Closed-loop temperature control performance of the microfluidic chip under different ambient temperatures: conventional strategy vs. proposed GWO-LSTM strategy. (**a**) Conventional strategy; (**b**) Proposed GWO-LSTM strategy.

To investigate the environmental adaptability of the proposed control strategy, different ambient temperatures (e.g., 10 °C, 20 °C, and 30 °C) were configured, while other experimental conditions were kept constant (initial chip temperature: 10 °C; initial thermal substrate temperature: 20 °C; maximum heating film voltage: 24 V). The simulation results demonstrate that, under the proposed temperature control strategy, the settling time was less than 120 s and the steady-state error of the reaction solution temperature remained below 0.1 °C under all tested environmental conditions, as illustrated in Figure 6b.

In contrast, when a conventional temperature control strategy for microfluidic chips was adopted, the settling time was significantly prolonged, and the steady-state error of the reaction solution temperature exceeded 1 °C in some cases, surpassing the ±2% tolerance band, as shown in Figure 6a.

These findings confirm that the proposed temperature control method achieves rapid and stable temperature regulation under varying ambient temperatures, featuring a short adjustment time and minimal steady-state error. Its overall performance fully meets the operational requirements of microfluidic chip-based immunoassay testing.

Experimental validation was conducted under three constant ambient temperature conditions (7 °C, 18 °C, and 28 °C), using microfluidic chips that had been equilibrated to each respective environmental temperature. Two temperature control strategies were comparatively evaluated: (1) the conventional thermal substrate feedback control method and (2) the fuzzy PID control strategy based on GWO-LSTM-estimated temperature feedback. Throughout the entire experimental process, temperature data from both the reaction solution and the thermal substrate were sampled at 1 s intervals and recorded.

In these temperature control validation experiments, the inserted temperature sensor served only as an independent reference recorder for evaluating the actual thermal response of the reaction solution. The real-time control input of the proposed strategy remained the virtual temperature estimated by the embedded GWO-LSTM model, while the conventional strategy used the thermal substrate temperature feedback.

**Figure 7:**
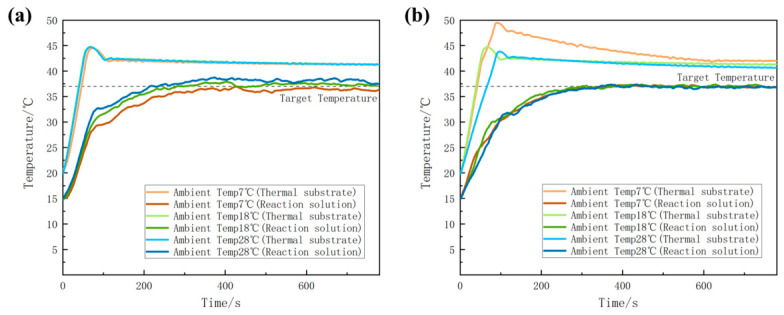
Microfluidic chip temperature control: conventional strategy vs. proposed GWO-LSTM-based fuzzy PID strategy. (**a**) Conventional strategy; (**b**) Proposed strategy.

When the conventional temperature control method for microfluidic chips was employed, with a ±2% error band as the evaluation criterion, the settling time required was up to approximately 330 s. Furthermore, the settling time varied by more than 100 s under different ambient temperature conditions, and the steady-state error of the reaction solution temperature reached as high as 0.6 °C, as shown in Figure 7a.

By contrast, when the temperature control strategy proposed in this study was adopted, the settling time was reduced to approximately 270 s and the steady-state error of the reaction solution temperature consistently remained below 0.2 °C, as illustrated in Figure 7b.

The data presented in the table indicate that, under varying ambient temperatures, the fuzzy PID control system based on GWO-LSTM-estimated temperature feedback achieved a significantly lower steady-state error and enhanced robustness compared with the conventional approach. In addition, the proposed method shortened the settling time by 18.2% relative to the traditional temperature control strategy; these results are summarized in Table 5.

### 3.3. Integrated Closed-Loop Control Performance Under Different Initial Chip Temperatures

To evaluate the control stability of the proposed temperature control strategy under different initial chip temperatures, closed-loop performance simulations were additionally conducted in the Python environment. The initial chip temperature was set to 5 °C, 10 °C, and 15 °C, respectively, while other experimental conditions remained unchanged (ambient temperature 20 °C, initial temperature of the thermal substrate 20 °C, and maximum heating film voltage 24 V).

**Figure 8:**
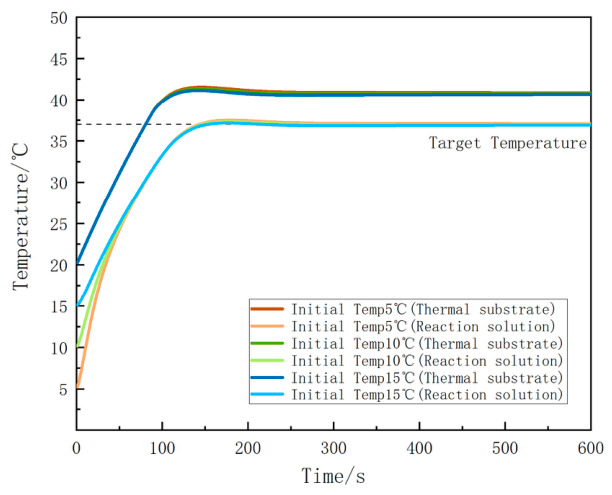
Stability of the proposed control strategy under different initial chip temperatures.

The simulation results demonstrate that the proposed temperature control method enabled the reaction solution temperature within the microfluidic chip to rapidly and stably reach the target temperature under all tested initial conditions. The settling time was less than 120 s and the steady-state error remained below 0.1 °C, which indicates strong control robustness. As shown in Figure 8, the proposed strategy effectively eliminates the influence of variations in the initial chip temperature on the regulation of the reaction solution temperature.

To simulate different storage conditions and evaluate the system’s capability to rapidly and stably reach the preset incubation temperature from various initial states, comparative temperature control experiments were conducted under different initial chip temperatures to assess the dynamic response performance of the system.

The microfluidic chips and their associated modules were first placed in low-temperature environments of 5 °C, 10 °C, and 15 °C, respectively, and maintained until thermal equilibrium was achieved. Subsequently, the incubation system was quickly activated, with the target temperature set to 37 °C. At a room temperature of 20 °C, temperature control experiments were carried out on the microfluidic chip using two distinct strategies: the conventional control method based on thermal substrate temperature feedback and the fuzzy PID control method based on GWO-LSTM estimated temperature feedback. Temperature data were sampled at 1 s intervals, and the temperatures of both the reaction solution and the thermal substrate were recorded throughout the entire experimental process.

As in the ambient temperature validation experiments, the reaction solution sensor in this experiment was inserted only for offline performance assessment and was not connected to the control feedback loop. This arrangement allowed the actual reaction solution temperature to be recorded for comparison while preserving the practical control logic based on non-contact virtual sensing.

**Figure 9:**
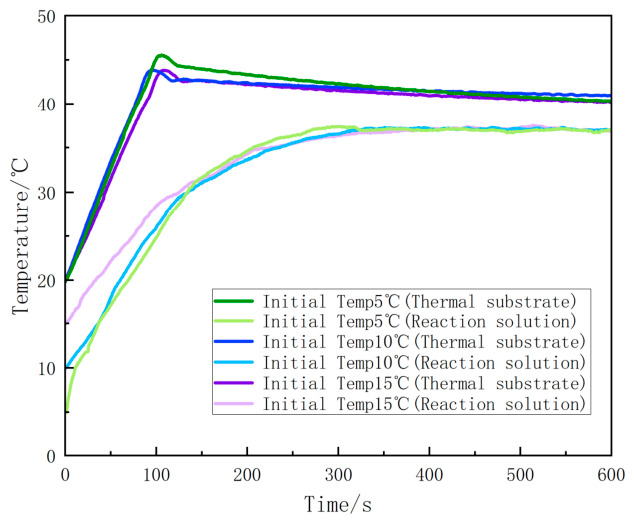
Dynamic response of the proposed temperature control strategy under different initial chip temperatures.

When the proposed temperature control method was adopted, the settling time was less than 290 s and the steady-state error of the reaction solution temperature remained below 0.2 °C, as illustrated in Figure 9. The results demonstrate that the fuzzy PID control system based on GWO-LSTM-estimated feedback exhibited strong control robustness under different initial chip temperatures.

### 3.4. Impact on Chemiluminescence Immunoassay Performance

Under three constant ambient temperatures (7 °C, 18 °C, and 28 °C), complete cardiac troponin I (cTnI) chemiluminescence assays were performed to compare two temperature control strategies: the conventional control method based on thermal substrate temperature feedback and the fuzzy PID control method integrated with GWO-LSTM-estimated temperature feedback.

In the reaction chamber, 50 μL of antibody-labeled magnetic beads, 50 μL of antibody-labeled alkaline phosphatase (ALP), and 10 μL of cTnI antigen (at a concentration of S4) were added sequentially. Each washing chamber was filled with 100 μL of washing buffer, while 40 μL of APS-5 substrate solution was introduced into the detection chamber. The system was incubated in the incubation module for 5 min, followed by magnetic separation, washing procedures, and subsequent optical detection and counting. To ensure experimental consistency, identical microfluidic chips and reagents were used at each time point, and the final chemiluminescence (CL) intensity was recorded. A comparative experiment was conducted between the fuzzy PID control method using GWO-LSTM-estimated temperature feedback and the conventional control method using thermal substrate temperature feedback. For each method, experiments were performed five times at ambient temperatures of 7 °C, 18 °C, and 28 °C, resulting in a total of 30 experimental trials for subsequent analysis.

**Figure 10:**
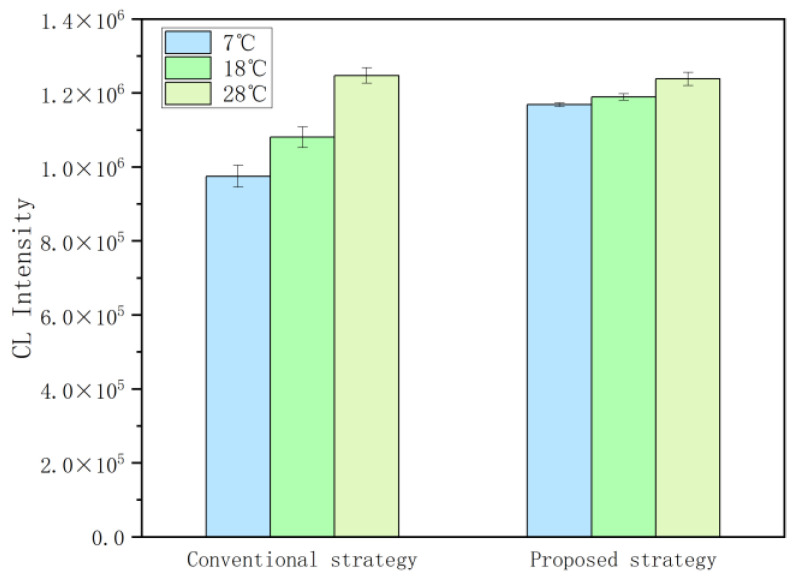
Comparison of chemiluminescence intensity in immunoassays under different temperature control strategies across various ambient temperatures.

The chemiluminescence values obtained under the two different temperature control strategies were directly compared to quantify the impact of temperature control accuracy on assay performance. Both the coefficient of variation (CV) and the mean chemiluminescence intensity were calculated to evaluate assay stability. As illustrated in Figure 10, the fuzzy PID control method based on GWO-LSTM-estimated feedback achieved a CV of no more than 2.69%, with a variation in chemiluminescence values of less than 4.27% across different ambient temperatures. In contrast, the conventional thermal substrate feedback control method resulted in a CV of 10.77%, accompanied by a chemiluminescence variation of approximately 15.78% under different ambient temperature conditions. These results clearly demonstrate that the proposed temperature control system significantly improved the stability of the incubation process, thereby enhancing the reliability of cTnI chemiluminescence assay results.

Chemiluminescence immunoassay (especially for cardiac troponin detection mentioned in this work) relies strictly on constant incubation temperature to guarantee consistent antigen–antibody binding efficiency. When temperature fluctuation reaches 0.6 °C (conventional method in Table 5), the binding rate of immune complexes will produce obvious deviation, leading to significant inter-batch deviation of luminescence signal intensity. For low-concentration clinical samples near the critical cut-off value of myocardial injury, such temperature drift may cause false positive or false negative diagnosis results, seriously interfering with accurate clinical judgment. When the temperature error is controlled within 0.2 °C (our method in Table 5), the fluctuation of immune reaction efficiency is suppressed to an acceptable range specified by clinical diagnostic standards. The repeatability and linearity of luminescence signals are greatly improved, effectively eliminating misdiagnosis risks caused by temperature drift and meeting the high-precision detection demand of trace biomarkers in point-of-care clinical scenarios.

We have collected the public technical specifications of mainstream commercial fully automatic chemiluminescence analyzers on the market for comparison. Most bench-top commercial instruments adopt semiconductor heating/cooling modules, with a nominal temperature control accuracy of ±0.2~0.5 °C under stable laboratory ambient conditions; under complex on-site clinical environments with large ambient temperature variations, their actual temperature drift often exceeds 0.4 °C.

The proposed temperature control strategy in this work can maintain the temperature error stably below 0.2 °C under wide ambient temperature disturbance. Our temperature stability performance is comparable to the high-end commercial chemiluminescence platforms and shows obvious advantages in anti-interference capability for variable clinical ambient temperatures, which proves the practical application potential of the microfluidic detection system developed in this paper for clinical on-site diagnosis.

## 4. Conclusions

This study targets the poor temperature control accuracy of the incubation unit in microfluidic chemiluminescence immunoassay systems and proposes an optimized scheme that combines multiphysics simulation, intelligent temperature estimation and fuzzy PID control. Finite element simulation was adopted to analyze the thermal coupling behavior between the heating substrate and reaction solution. The GWO-LSTM model was adopted to realize high-precision temperature estimation of the reaction solution, and closed-loop control was further verified on the actual device. Experimental results showed that the developed strategy effectively strengthened the thermal regulation performance of the incubation module. Benefiting from accurate temperature compensation and stable dynamic regulation, the coefficient of variation in cTnI detection was significantly reduced and the overall repeatability and stability of chemiluminescence immunoassays were greatly enhanced. Compared with conventional indirect temperature control methods, the developed strategy exhibits obvious advantages in anti-interference capability under variable ambient and initial conditions. This work provides a reliable engineering reference for the performance optimization of integrated biochemical detection platforms. In future research, the algorithm operation efficiency and hardware integration level will be further optimized to adapt to more compact and low-power portable detection equipment.

## Figures and Tables

**Table 1:** Materials, density, thermal conductivity, and heat capacity of each component in the model.

Model	Material	Density (kg/m^3^)	Heat Conductivity (W/(m∙K))	Heat Capacity (J/(kg∙K))
Microfluidic chip	PTFE	2200	0.24	1050
Thermal substrate	Aluminum	2710	237	902
Heating films	Nichrome	9000	15	500
Reaction solution domain	Water	1000	0.57	4200
	Paraffin Oil	827	0.3	1870

**Table 2:** Error between the simulated and experimental heating results.

Model	MAPE	R^2^
Reaction solution	3.46%	95.65%
Thermal substrate	4.82%	92.97%

**Table 3:** GWO hyperparameter optimization results.

Hyper-Parameter	Optimized Result
Hidden size	128
Number of layers	1
Dropout	0.1959
Learning rate	0.000246

**Table 4:** Model optimization results.

Method	GWO-LSTM	LSTM
RMSE	0.17 °C	0.35 °C
MAE	0.13 °C	0.30 °C
R^2^	99.64%	93.86%

**Table 5:** Comparison of temperature control accuracy under different ambient temperatures.

	Method	Settling Time (Conventional Method)/s	Steady-State Error (Conventional Method)/°C	Adjusting Time (Proposed)/s	Steady-State Error (Proposed)/°C
Ambient Temperature					
7 °C		330	0.67	267	0.21
18 °C		240	0.13	257	0.18
28 °C		201	0.56	268	0.18

## Data Availability

Dataset available on request from the authors.
